# Prognostic Value of Pre-operative Renal Insufficiency in Urothelial Carcinoma: A Systematic Review and Meta-Analysis

**DOI:** 10.1038/srep35214

**Published:** 2016-10-11

**Authors:** Jian Cao, Xiaokun Zhao, Zhaohui Zhong, Lei Zhang, Xuan Zhu, Ran Xu

**Affiliations:** 1 Department of Urology, The Second Xiangya Hospital, Central South University, 139 Middle Renmin Road, Changsha, Hunan 410011, P.R. China; 2 MRC Centre for Reproductive Health, Queen’s Medical Research Institute, 47 Little France Crescent, Edinburgh EH16 4TJ, United Kingdom

## Abstract

The effect of pre-operative renal insufficiency on urothelial carcinoma (UC) prognosis has been investigated by numerous studies. While the majority report worse UC outcomes in patients with renal insufficiency, the results between the studies differed wildly. To enable us to better estimate the prognostic value of renal insufficiency on UC, we performed a systematic review and meta-analysis based on the published literature. A total of 16 studies which involved 5,232 patients with UC, investigated the relationship between pre-operative renal insufficiency and disease prognosis. Estimates of combined hazard ratio (HR) for bladder urothelial carcinoma recurrence, cancer-specific survival (CSS) and overall survival (OS) were 1.65 (95% CI, 1.11–2.19), 1.59 (95% CI, 1.14–2.05) and 1.45 (95% CI, 1.19–1.71), respectively; and for upper urinary tract urothelial carcinoma recurrence, CSS and OS were 2.27 (95% CI, 1.42–3.12), 1.02 (95% CI, 0.47–1.57) and 1.52 (95% CI, 1.05–1.99), respectively. Our results indicate that UC patients with pre-operative renal insufficiency tend to have higher recurrence rates and poorer survival compared to those with clinically normal renal function, thus renal function should be closely monitored in these patients. The impact of intervention for renal insufficiency on the prognosis of UC needs to be further studied.

Urothelial carcinoma (UC) is ranked as the fourth most common tumour in the United States and Europe^1^, they are a heterogeneous group of cancers that arise from the transitional epithelium of the lower (bladder and urethra) or the upper (pyelocaliceal cavities and ureter) urinary tract. Bladder cancer accounts for 90–95% of UC^1^, and is the 11^th^ most commonly diagnosed cancer, and the 14^th^ leading cause of cancer deaths worldwide^2^. In contrast, upper urinary tract urothelial carcinoma is rare and accounts for only 5–10% of UC^1^. Among all patients with upper urinary tract urothelial carcinoma, 17% are diagnosed with synchronous bladder cancer; 22–47% experience recurrence of bladder cancer; and 2–6% experience recurrence of cancer in the contralateral upper tract^1^. On the other hand, patients with primary bladder cancer are at low risk (0.7%) of developing subsequent upper urinary tract urothelial carcinoma^3^.

Higher incidences of malignancies have been observed in patients with chronic renal failure^4^,^5^. In a prospective cohort study conducted in Finnish male smokers, Stephanie *et al*. found that serum creatinine was positively associated with significantly greater risk of prostate cancer (odd ratio = 2.23, *p* = 0.0008; highest versus lowest quartile)^6^. In a large cohort study conducted in patients with renal cell carcinoma, Solomon *et al*. found that lowered renal function was independently associated with an increased likelihood of papillary renal cell carcinoma histology^7^. There is also increasing evidence to suggest that pre-operative renal function may indicate urological cancer prognosis: previous studies have identified renal insufficiency as a risk factor for cancer recurrence in prostate cancer^8^, bladder cancer^9^, and upper urinary tract urothelial carcinoma^10^. However, there has been no consensus on the prognostic value of pre-operative renal insufficiency in UC. With the aim of deriving a more precise estimate of the prognostic value of pre-operative renal insufficiency, we performed a systematic review of the published studies and used standard meta-analysis techniques to pool the available evidence and summarize them quantitatively.

## Results

### Characteristics of Included Studies

**Figure 1:**
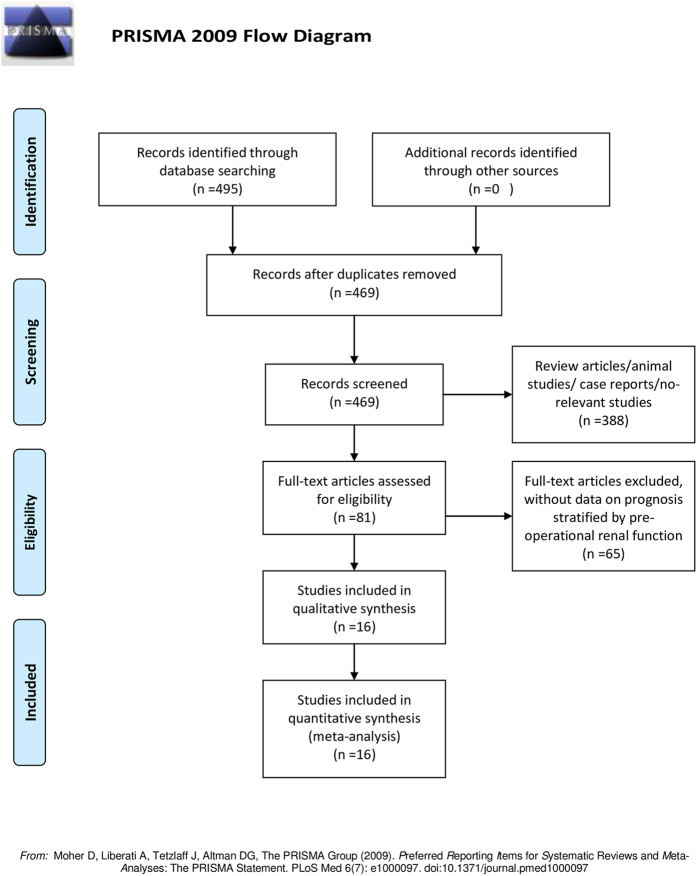
Flow diagram of literature search and selection for meta-analysis.

The flow chart of the literature search is shown in Fig. 1. The initial search yielded a total of 495 potentially relevant abstracts, among which 16 articles^9^,^10^,^11^,^12^,^13^,^14^,^15^,^16^,^17^,^18^,^19^,^20^,^21^,^22^,^23^,^24^ met all inclusion criteria. A total of 5,232 patients with UC were included in this meta-analysis. Patients were enrolled from secondary care departments. The subtypes of UC included for study were upper urinary tract urothelial carcinoma (n = 8), or bladder urothelial carcinoma (n = 8). All 16 included studies were retrospective, seven studies presented recurrence rates, and six presented data of OS and CSS. The characteristics of the included studies are summarized in Tables 1 and 2 and Supplemental Table 1. For renal function classification, 11 studies used serum creatinine, and five used estimated glomerular filtration rate (eGFR). Quality assessment analysis ranked 11 studies as “excellent quality”, and the remaining studies as “good quality” (Table 1).

In one study by Chung *et al*.^16^, patients were divided into three groups: absence of chronic kidney disease (CKD), stage 1–4 CKD, and stage 5 CKD. In order to reduce heterogeneity, we combined data of No CKD vs. stage 1–4 CKD. In a study by Li *et al*.^19^, odd ratios of contralateral upper urinary tract urothelial carcinoma recurrence, bladder cancer recurrence, and local recurrence were all reported, and the data of local recurrence was choose which was most relevant.

### The Prevalence of Pre-operative Renal Insufficiency in Urothelial Carcinoma

Eighteen studies here^9^,^13^,^16^,^17^,^18^,^19^,^20^,^21^,^22^,^23^,^24^,^25^,^26^,^27^,^28^,^29^,^30^,^31^ were used to estimate the prevalence of renal insufficiency in patients with UC (Table 2). The prevalence of pre-operative renal insufficiency in UC in this systematic review was 35.9% (ranging from 11.9% to 75.3%). The prevalence of pre-operative renal insufficiency in studies of bladder cancer was 16.9% (ranging from 13.0% to 25.5%). The prevalence of pre-operative renal insufficiency in studies of upper urinary tract urothelial carcinoma was 44.9% (ranging from 16.8% to 75.3%).

### Association of Pre-operative Renal Insufficiency with Prognosis of Bladder Urothelial Carcinoma

#### Recurrence

**Figure 2:**
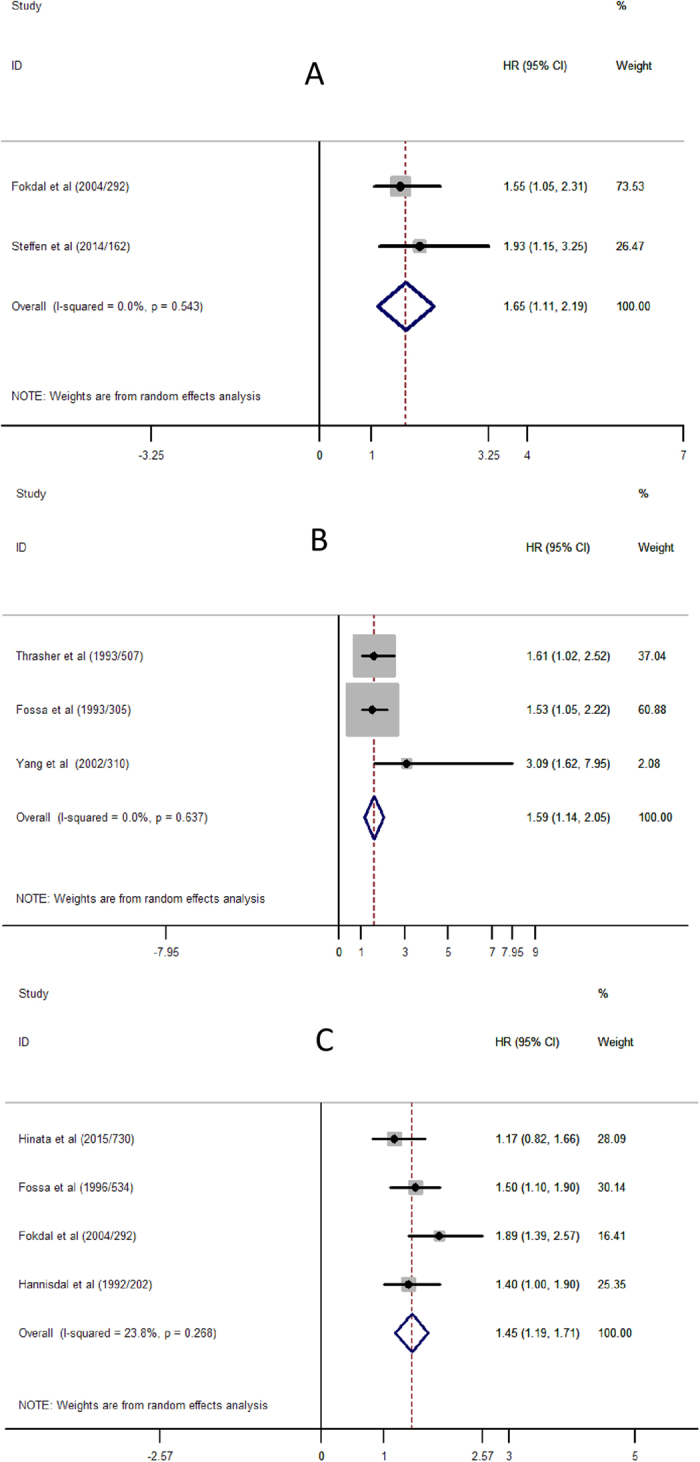
Meta-analysis of the association between renal insufficient and prognosis of Bladder Urothelial Carcinoma. (**A**) Recurrence for Bladder Cancer; (**B**) Cancer-specific Survival for Bladder Cancer; (**C**) Overall Survival for Bladder Cancer. Each study was shown by the name of the first author (publish year/patients numbers) and the HRs with 95% CIs.

The combined recurrence data showed that pre-operative renal insufficiency was associated with an increase disease recurrence (HR = 1.65; 95% CI, 1.11 to 2.19) (Fig. 2A).

#### Cancer-specific Survival

The pooled HR of pre-operative renal insufficiency for bladder urothelial carcinoma CSS was 1.59 (95% CI, 1.14 to 2.05) (Fig. 2B).

#### Overall survival

The pooled HR of pre-operative renal insufficiency for OS was 1.45 (95% CI, 1.19 to 1.71) (Fig. 2C).

### Association of Pre-operative Renal Insufficiency with Prognosis of Upper Urinary Tract Urothelial Carcinoma

#### Recurrence

**Figure 3:**
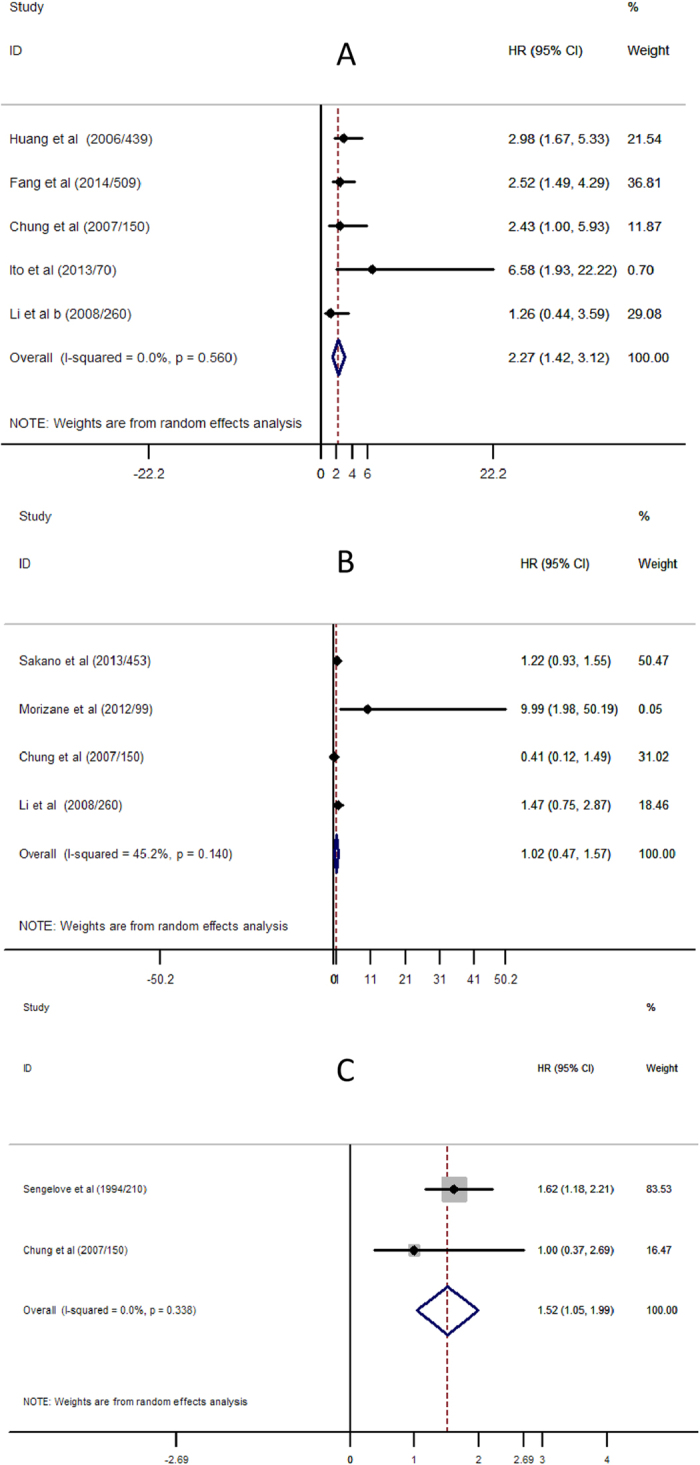
Meta-analysis of the association between renal insufficient and Prognosis of Upper Urinary Tract Urothelial Carcinoma. (**A**) Recurrence for Upper Urinary Tract Urothelial Carcinoma; (**B**) Cancer-specific Survival for Upper Urinary Tract Urothelial Carcinoma; (**C**) Overall Survival for Upper Urinary Tract Urothelial Carcinoma. Each study was shown by the name of the first author (publish year/patients numbers) and the HRs with 95% CIs.

The combined recurrence data showed that pre-operative renal insufficiency was associated with an increased disease recurrence, with the HR of renal insufficiency being 2.27 (95% CI, 1.42 to 3.12) for recurrence of upper urinary tract urothelial carcinoma (Fig. 3A). In a secondary subgroup analysis of recurrence of upper urinary tract urothelial carcinoma stratified by renal function testing methods, the HR of pre-operative renal insufficiency was 2.05 (95% CI, 0.37 to 3.73) when using serum creatinine test, and 2.56 (95% CI, 1.35 to 3.76) when using eGFR test (Supplemental Figure 1). In another secondary subgroup analysis stratified by recurrence location, pre-operative renal insufficiency was identified as a risk factor (HR = 2.69; 95% CI, 1.58 to 3.80) for recurrence in contralateral upper urinary tract, the risk for recurrence in bladder was similar but not statistically significant (HR = 2.43; 95% CI, −0.03 to 4.89) (Supplemental Figure 2).

#### Cancer-specific Survival

The pooled HR of pre-operative renal insufficiency for CSS was 1.02 (95% CI, 0.47 to 1.57) (Fig. 3B).

#### Overall Survival

The pooled HR of pre-operative renal insufficiency for OS was 1.52 (95% CI, 1.05 to 1.99) (Fig. 3C).

## Discussion

Although renal insufficiency is not uncommon in patients with UC, the influence of pre-operative renal insufficiency on cancer prognosis has not received significant attention. In this systematic review, 16 eligible studies were included to investigate the relationship between pre-operative renal insufficiency and disease recurrence, CSS and OS of UC. Our study shows that the prevalence of pre-operative renal insufficiency is high in UC patients, especially in patients with upper urinary tract urothelial carcinoma, and pre-operative renal insufficiency is significantly related to higher disease recurrence rates and worse OS for both bladder and upper urinary tract urothelial carcinoma patients, but only significantly associated with worse CSS for bladder urothelial carcinoma patients.

The prevalence of pre-operative renal insufficiency in UC patients in this meta-analysis is 36.1% (16.9% in bladder cancer and 44.9% in upper urinary tract urothelial carcinoma patients). It is reported that the prevalence of renal insufficiency is high among cancer patients. In a pilot study conducted by Vincent *et al*. one third of the included 316 cancers patients had pre-operative renal insufficiency^32^. A later study conducted by the same research group among 4,684 cancer patients from 15 centres showed more than half of the cancer patients had pre-operative renal insufficiency^33^. UC patients often have a complex constellation of risk factors for renal impairment such as advanced age, synchronous urinary tract obstruction/infection and other comorbidities. The higher frequency of pre-operative renal insufficiency in patients with upper urinary tract urothelial carcinoma may be explained in part by the higher frequency of ureteral obstruction observed in these patients than in those patients with bladder cancer. It should be noted that the diagnosis of renal insufficiency may vary between studies included in this meta-analysis because of the different testing methods and cutoff values which were used. Currently, serum creatinine is the most commonly used testing method for renal function but it is dependent on muscle mass, and as such the alternative testing methods such as creatinine clearance or eGFR (using either the Cockcroft-Gault formula or the abbreviated Modification of Diet in Renal Disease formula) are suggested to be more accurate than serum creatinine^33^. This may explain that in our meta-analysis, the risk factors of pre-operative renal insufficiency for recurrence of upper urinary tract urothelial carcinoma were similar when calculated using eGFR value (HR = 2.56) and using serum creatinine (HR = 2.05), but the latter failed to reach statistical significance due to great between-study variability.

Our study suggests that for patients with UC, pre-operative renal insufficiency is associated with higher disease recurrence (HR = 1.65 for bladder urothelial carcinoma; HR = 2.27 for upper urinary tract urothelial carcinoma), poorer OS (HR = 1.45 for bladder urothelial carcinoma; HR = 1.52 for upper urinary tract urothelial carcinoma), and worse CSS for patients with bladder urothelial carcinoma (HR = 1.59). Renal insufficiency is a spectrum of diseases that can induce multisystem dysfunction. Renal insufficiency causes impaired ability to maintain fluid and electrolyte homeostasis, and consequently causes a potential for the accumulation of toxic metabolic products in the internal milieu^34^. Renal insufficiency usually causes less urine production and delayed haematuria presentation, both of which may delay disease diagnosis^35^. Most patients in our study had undergone operations which could cause further deterioration of renal function, particularly in those with pre-operative renal insufficiency^36^. In addition, most cancer therapy agents are cleared through the kidney and are nephrotoxic, including chemotherapy agents, molecular targeted agents, pain management agents, radiopharmaceuticals, and contrast agents used in radiology^37^. Therefore, impaired renal function not only leads to pathophysiological changes but also greatly restricts treatment options; for example, patients with a creatinine clearance of <60 ml/min are ineligible for cisplatin treatment. Gupta N *et al*. found that ileal conduit may not be ideal for patients with serum creatinine greater than 2.5 mg/dl^38^. To avoid severe renal toxicities caused by chemotherapeutic agents with nephrotoxic properties, drug dosages are usually reduced in patients with renal insufficiency, which may lead to sub-optimal drug concentrations and compromise treatment outcomes.

Efforts have been made to reveal the molecular mechanism underlying the association between pre-operative renal insufficiency and poor prognosis of UC. Immune defect is the most commonly mentioned mechanism; for example, patients with uremia usually show a state of immune dysfunction characterized by immunosuppression (such as impaired T lymphocyte activation, increased Th1/Th2 ratio, decreased B lymphocyte count, reduced ability of antigen-presenting cells to recognize tumour-associated antigens, hypo-reactive monocytes, and decreased bactericidal abilities of neutrophils^39^) thereby fostering tumour progression. An increased prevalence of inflammation was observed in patients with moderate to severe CKD^40^, and it is well established that inflammation plays a critical role in tumour promotion and progression. Impaired DNA repair ability, nutritional deficiencies, and chronic urinary tract infection are also plausible explanations for the association between renal insufficiency and poorer UC prognosis^41^,^42^.

In a subgroup analysis stratified by recurrence location of upper urinary tract urothelial carcinoma, pre-operative renal insufficiency was a risk factor for recurrence in contralateral upper urinary tract (HR = 2.69), although the risk for recurrence in bladder was similar, it did not reach statistical significance. The difference in HR may ascribe to the greater aggressiveness of upper urinary tract urothelial carcinoma compared with bladder cancer. This may also explain why pre-operative renal insufficiency did not seem to influence the CSS in patients with upper urinary tract urothelial carcinoma; unlike in patients with bladder cancer. But the lack of association between pre-operative renal insufficiency and CSS in patients with upper urinary tract urothelial carcinoma could be because such survival analyses usually have short follow-up time (probably because upper urinary tract urothelial carcinoma tend to have early recurrence), therefore, we suggest that more studies are needed to discover whether there is association between pre-operative renal insufficiency and a longer term survival rate in patients with upper urinary tract urothelial carcinoma.

There are several limitations in this study. Firstly, all included data were from retrospective observational studies; secondly, the effect sizes of prognosis aggregated in this meta-analysis were obtained from multivariate or univariate Cox/regression analysis, and some were reconstructed from survival curves. Finally, our meta-analysis is limited by the lack of universal testing method and classification for renal insufficiency between the studies — either serum creatinine or eGFR were used to indicate renal function, and the cutoff values for same testing methods also varied between studies, particularly when the serum creatinine value was used.

Notwithstanding these limitations, our meta-analysis collected data from more than 5,000 UC cases, and offers a comprehensive overview of the existing evidence for the correlation between pre-operative renal insufficiency and worse prognosis of UC, which suggests that clinicians should take renal function into careful account when treating UC patients. However, whether, and to what extent, treating UC patient’s renal insufficiency affects their prognosis still needs further study.

## Methods

### Design

This systematic review and meta-analysis was conducted according to the Preferred Reporting Items for Systematic Reviews and Meta-analysis (PRISMA)^43^. A review protocol consisting of Background, Review, Objectives, and Methodology was established prior to conducting the review (Supplemental File 1). The protocol was followed rigorously for each phase of the review. The presentation of results follows the PRISMA-statement (Supplemental File 2).

### Search Strategy and Study Selection

Systematic literature searches of PubMed, the Cochrane Library, the China National Knowledge Infrastructure, and EMBASE (including American Urological Association and European Association of Urology meeting abstracts) were performed to identify potentially relevant studies; the last search was on February 20, 2016. We used the keywords “urothelial cancer”, “transitional cell cancer”, combined with terms related to renal insufficiency, including “renal insufficiency”, “eGFR”, “serum creatinine”, “renal function”, and “prognosis”. Studies were eligible if prognosis was analysed in UC cases stratified by pre-operative renal function. The primary outcomes of interest were disease recurrence, OS, and CSS. Studies which investigated only post-operative renal function were excluded.

All the studies from PubMed, and EMBASE which met the inclusion criteria were retrieved using a search filter from McMaster University of Health Information Research Unit for the best balance of sensitivity and specificity for prognostic studies. (http://hiru.mcmaster.ca/hiru/HIRU_Hedges_MEDLINE_Strategies.aspx). Review articles and bibliographies of other relevant studies identified were hand-searched to find additional studies. Unpublished data and data from review articles, case reports, abstracts, and letters were not included. Data were extracted using a reporting checklist proposed by the Meta-analysis of Observational Studies in Epidemiology (MOOSE) Group^44^. Two authors independently selected studies and performed data extraction, and any discrepancies were resolved by consensus.

### Assessment of Publication Quality

Quality assessment analysis following the method for review of prognostic tests proposed by the US Department of Health and Human Services was performed for each study included in our meta-analysis^45^. The assessment system (Supplementary File 3) consists of 10 questions, each of which used an ordinal scale with possible values of 0, 1, and 2, where 0 represented “not clear” or “not a relevant item” and higher values indicated higher quality. Two independent investigators assessed each study using this system and a consensus was reached through discussion. Final scores were represented as a percentage. Scoring >75% was considered “excellent quality”; scoring between 50–70% was “good quality” and, scoring <50% was considered “poor quality.”

### Statistical Analysis

Standard meta-analysis methods^46^ were applied to evaluate the overall effect of renal function on the prognosis of UC. For studies performing only univariate survival analysis (comparison of Kaplan-Meier survival curves based on the log-rank test), HR and 95% CI were calculated from survival curves adopting a hierarchical series of steps according to Tierney *et al*.^47^. Given that the statistical methods (log-rank and Cox model) used by studies included in our meta-analysis were inconsistent, results were combined using the generic inverse variance method^46^. Due to a priory of assumptions about the likelihood of heterogeneity between primary studies, we performed our meta-analysis using the random-effects model which is usually more conservative. Sensitivity to influential studies was evaluated by the “leave-one-out” cross-validation procedure whereby we omitted every study from the meta-analysis consecutively and recalculated of the remaining pooled HRs. A subgroup analysis considering more homogeneous sets of studies (i.e., those using the same stratification and study design feature) was adopted as an additional sensitivity test.

### Publication Bias

Publication bias (linked to the fact that negative trials are cited less frequently and are therefore more likely to be missed in the search for relevant studies) of studies was analysed using the Begg’s funnel plot and the Egger’s linear regression test. Unless otherwise noted, no significant heterogeneity or publication bias was marked among included studies. All statistical analyses were conducted using software STATA version 12.0 (STATA Corporation, College Station, TX, USA). All statistical tests were two-sided and the significance level was set at 5%.

## Additional Information

**How to cite this article**: Cao, J. *et al*. Prognostic Value of Pre-operative Renal Insufficiency in Urothelial Carcinoma: A Systematic Review and Meta-Analysis. *Sci. Rep*. **6**, 35214; doi: 10.1038/srep35214 (2016).

## Supplementary Material

Supplementary Information

Supplementary Table S1

## Figures and Tables

**Table 1:** Characteristics of 16 eligible studies for meta-analysis.

Study	Number	Area	Research Time	Location^1^	Quality score (%)^2^	End Points^3^	RI Indicator^4^	Reference
Steffen (2014)	162	Germany	1996–2006	Ba	80%	Re	eGFR (<60 vs. >60 ml/min)	9
Hinata (2015)	730	Japan	2001–2010	Ba	85%	OS	eGFR (<60 vs. >60 ml/min)	11
Fossa (1996)	534	Norway	1980–1991	Ba	65%	OS	SrC (≥120 vs. <120 μmol/l)	12
Fokdal (2004)	292	Denmark	1994–2002	Ba	80%	OS/Re	SrC (≥120 vs. <120 μmol/l)	13
Hannisdal (1993)	202	Norway	1980–1987	Ba	55%	OS	SrC (≥120 vs. <120 μmol/l)	14
Thrasher (1993)	507	USA	1969–1990	Ba	55%	CSS	SrC (≥1.5 vs. <1.5 mg/dl)	20
Fossa (1993)	305	Norway	1980–1990	Ba	50%	CSS	SrC (≥150 vs. <150 μmol/l)	23
Yang (2002)	310	Taiwan	1987–1997	Ba	85%	CSS	SrC (>3 vs. <1.5 mg/dl)	24
Sengeløv (1994)	210	Demark	1976–1991	UUT	65%	OS	SrC (normal vs. <abnormal)	15
Chung (2007)	150	Taiwan	1996–2006	UUT	75%	OS/Re/CSS	No CKD vs. Earlier CKD	16
Huang (2006)	439	Taiwan	1977–2003	UUT	80%	Re	SrC (≥2.0 vs. <2.0 mg/ml)	17
Fang (2014)	509	China	2000–2010	UUT	85%	Re	eGFR (<60 vs. >60 ml/min)	10
Ito (2013)	70	Japan	1999–2012	UUT	75%	Re	eGFR <60 vs. >60 ml/min)	18
Li (2008)	260	Taiwan	1990–2005	UUT	75%	Re	SrC (>1.4 vs. ≤1.4 mg/dl)	19
Sakano (2013)	453	Japan	1995–2009	UUT	75%	CSS	SrC (≥1.3 vs. <1.3 mg/dl)	21
Morizane (2012)	99	Japan	1995–2011	UUT	75%	CSS	SrC (≥1.0 vs. <1.0 mg/dl)	22

^1^Ba-bladder; UUT-upper urinary tract.

^2^percentages of total possible score.

^3^OS-overall survival; CSS-cancer specific survival; Re-recurrence.

^4^RI-renal insufficiency; eGFR-estimated glomerular filtration rate; SrC-serum creatinine; CKD-chronic kidney disease.

**Table 2:** The incidences of renal insufficiency in subgroups.

Study	Location^1^	Fit	Unfit	Total	RI%	RI Indicator^2^	Reference
Fokdal (2004)	Ba	237	55	292	18.84%	SrC (>120 μmol/l vs. ≤120 μmol/l)	13
Fossa (1993)	Ba	263	42	305	13.77%	SrC (>150 μmol/l vs. ≤150 μmol/l)	23
Pollack (1995)	Ba	194	29	223	13.00%	SrC (>1.5 mg/dl vs. ≤1.5 mg/dl)	25
Steffen (2014)	Ba	133	29	162	17.90%	eGFR (>60 ml/min vs. ≤60 ml/min)	9
Spera (1988)	Ba	100	16	116	13.79%	SrC (>1.5 mg/dl vs. ≤1.5 mg/dl)	26
Thrasher (1993)	Ba	433	74	507	14.59%	SrC (>1.5 mg/dl vs. ≤1.5 mg/dl)	20
Yang (2002)	Ba	231	79	310	25.48%	SrC (>1.5 mg/dl vs. ≤1.5 mg/dl)	24
***Subtotal***	***Ba***	***1591***	***324***	***1915***	***16.92%***		
Chen (2007)	UUT	40	64	104	61.54%	eGFR (>60 ml/min vs. ≤60 ml/min)	27
Chung (2007)	UUT	37	113	150	75.33%	No CKD vs. CDK stage 1–5	16
Fang (2015)	UUT	388	504	892	56.50%	eGFR (>60 ml/min vs. ≤60 ml/min)	28
Huang (2006)	UUT	349	90	439	20.50%	SrC (>2.0 mg/dl vs. ≤2.0 mg/dl)	17
Ito (2013)	UUT	39	31	70	44.29%	eGFR (>60 ml/min vs. ≤60 ml/min)	18
Li (2008)	UUT	158	102	260	39.23%	SrC (>1.4 mg/dl vs. ≤1.4 mg/dl)	19
Morizane (2012)	UUT	33	66	99	66.67%	SrC (>1.0 mg/dl vs. ≤1.0 mg/dl)	22
Sakano (2013)	UUT	377	76	453	16.78%	SrC (>1.3 mg/dl vs. ≤1.3 mg/dl)	21
Yafi (2014)	UUT	504	525	1029	51.02%	eGFR (>60 ml/min vs. ≤60 ml/min)	29
***Subtotal***	***UUT***	***1925***	***1571***	***3496***	***44.94%***		
Sengeløv (2000)	Mix	104	14	118	11.86%	SrC (Normal vs. High)	30
Ichioka (2015)	Mix	145	200	345	57.97%	eGFR (>60 ml/min vs. ≤60 ml/min)	31
***Subtotal***	***Mix***	***249***	***214***	***463***	***46.22%***		
***Total***	***ALL***	***3765***	***2109***	***5874***	***35.90%***		

SrC-serum creatinine; CKD-chronic kidney disease.

^1^Ba-bladder; UUT-upper urinary tract.

^2^RI-renal insufficiency; eGFR-estimated glomerular filtration rate.
