# Supplementary material for: Prognostic Value of Pre-operative Renal Insufficiency in Urothelial Carcinoma: A Systematic Review and Meta-Analysis
Source: Sci Rep. 2016 Oct 11;6:35214. doi: 10.1038/srep35214 (PMC5057078; doi:10.1038/srep35214)
Supplement: Supplementary Information [file srep35214-s1.docx]

**Prognostic Value of Pre-operative Renal Insufficiency** **in Urothelial Carcinoma：A Systematic Review and Meta-Analysis**

**Jian Cao^1, 2^, Xiaokun Zhao^1^，Zhaohui Zhong^1^, Lei Zhang^1^, Xuan Zhu^1^, and Ran Xu^1*^**

^1^ Department of Urology, The Second Xiangya Hospital, Central South University, 139 Middle Renmin Road, Changsha, Hunan 410011, P.R. China; ^2^MRC Centre for Reproductive Health, Queen’s Medical Research Institute, 47 Little France Crescent, Edinburgh EH16 4TJ, United Kingdom.

^*^ Corresponding author Ran Xu (Email: xuran@csu.edu.cn; Telephone: 0086 186-0841-8000; Fax Number: 0086 0731-85533525)

**Supplemental Figures 1-2**

**Supplemental Figure 1 Subgroup analysis of the association between renal insufficient and recurrence of UC stratified by renal function testing methods in the UUTUC setting.**


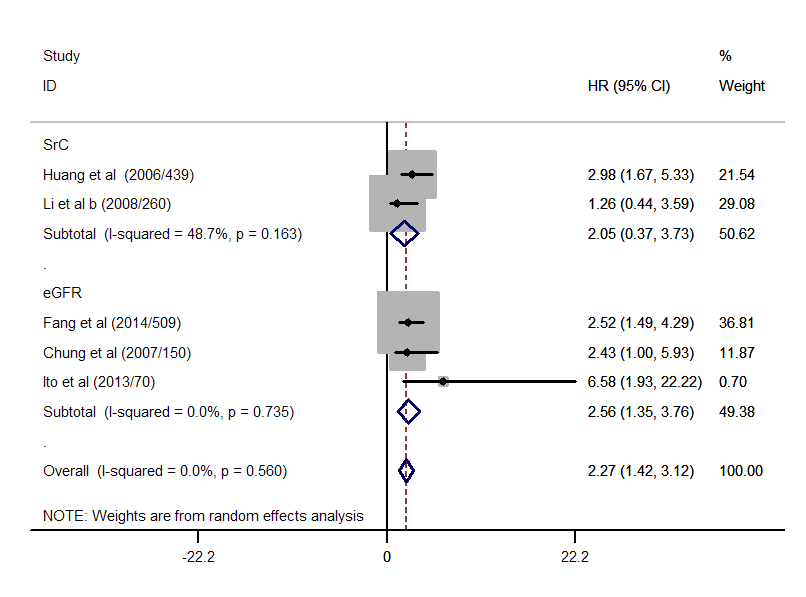


Each study was shown by the name of the first author (publish year/patients numbers) and the HR with 95%CIs. Subgroup analysis was shown according to renal function testing methods: SrC=Serum Creatinine; eGFR= estimated glomerular filtration rate.

**Supplemental Figure 2 Subgroup analysis of the association between renal insufficient and recurrence of UC stratified by recurrence locations in the UUTUC setting.**


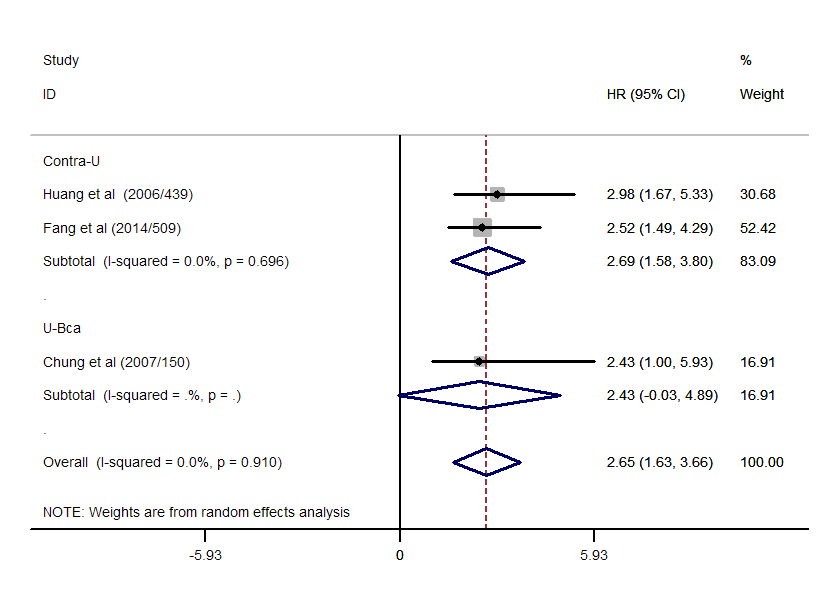


Each study was shown by the name of the first author (publish year/patients numbers) and the HR with 95%CIs. Subgroup analysis was shown according to recurrence locations: Con-UURe= contralateral UUTUC recurrence; BUC-Re= Bladder Urothelial Carcinoma recurrence.

| **Supplemental File 1 Systematic Review Protocol**  Review title and timescale | | |  |
| --- | --- | --- | --- |
| 1 Review title  Impact of Renal Insufficient on Urothelial Carcinoma Prognosis: A Systematic Review and Meta-Analysis | |  |  |
|  |  | |  |
|  |  | |  |
| Review team details | | |  |
| 2 | Organizational affiliation of the review The Second Xiangya Hospital, Central South University, China Website address: http://www.xyeyy.com/ | |  |
| 3 | Review team members and their organizational affiliations | |  |
|  | \| Title \| First name \| Last name \| Affiliation \| \| --- \| --- \| --- \| --- \| \| Dr \| Jian \| Cao \| The Second Xiangya Hospital, Central South University, China \| \| Dr \| ran \| Xu \| The Second Xiangya Hospital, Central South University, China \| \| Dr \| Xiaokun \| Zhao \| The Second Xiangya Hospital, Central South University, China \| \| Dr \| Yinhuai \| Wang \| The Second Xiangya Hospital, Central South University, China \| \| Dr \| Zhaohui \| Zhong \| The Second Xiangya Hospital, Central South University, China \| \| Dr \| lei \| Zhang \| The Second Xiangya Hospital, Central South University, China \| \| Dr \| Xuan \| Zhu \| The Second Xiangya Hospital, Central South University, China \| | |  |
| 4 | Funding sources/sponsors Jian Cao is sponsored by China Scholarship Council as a visiting researcher at the University of Edinburgh. | |  |
| 5 | Review question(s) A number of studies have investigated the influence of renal function on prognosis of patients with urothelial carcinoma (UC). Although most have reported worse prognosis with renal insufficient (RI), the results between studies differ wildly. To derive a more precise estimate of the prognostic significance of RI, we performed a meta-analysis based on published literature. | |  |
| **6** | Searches Systematic literature searches of PubMed, the Cochrane Library, China National Knowledge Infrastructure(CNKI), and EMBASE (including American Urological Association and European Association of Urology meeting abstracts) was performed to identify observational cohort studies analyzing the prognostic value of renal insufficient in patients with UC. We used the keywords “urothelial cancer”, “transitional cell cancer”, combined with terms related to renal insufficient, including “renal insufficient, eGFR, serum creatinine, renal function” and “prognosis”. Then a search filter from McMaster University of Health Information Research Unit was applied in retrieved results from PubMed, or EMBASE, for the sake of best balance of sensitivity and specificity for prognostic studies. (http://hiru.mcmaster.ca/hiru/HIRU_Hedges_MEDLINE_Strategies.aspx.) | |  |
|  |  | |  |
| 7 | Condition or domain being studied Urothelial carcinoma (UC) is the fourth most common tumour. Over 95% UC are derived from the urothelium and correspond to upper urinary tract tumour or bladder tumour. There is increasing evidence showing that RI can not only indicate general health condition but also the oncological outcomes in cancer patients. Patients with chronic renal failure have been reported to have an increased incidence of malignancies. | |  |
| 8 | Participants/population Studies were eligible if prognoses were analyzed in urothelial cancer patients stratified by renal function. The primary outcomes of interest were recurrence, overall survival, cancer-specific survival, and recurrence-free survival. Only studies providing information on prognosis were included; studies that investigate only oncological outcomes and post-operational renal function without prognosis analysis were excluded. | |  |
|  | Intervention(s), exposure(s) A number of studies have investigated the prognostic role of renal insufficiency (RI) in patients with urothelial cancer. Most of them reported RI as a potent prognostic factor in patients with urothelial cancer, but in the others, no significant association was found. Moreover, estimates of the prognostic value of RI between studies have differed wildly. | |  |
| Review methods | | |  |
| 9 | Types of study to be included There are restrictions on the types of study design as long as the studies reported prognosis data in urothelial cancer patients stratified by renal function. | |  |
| 10 | Primary outcome(s) Renal insufficiency is significantly associated with worse recurrence in urothelial cancer patients.  Effect measure: follow-up schedule | |  |
| 11 | Secondary outcomes Renal insufficiency is significantly associated with worse overall survival in urothelial cancer patients. | |  |
| 12 | Effect measure: follow-up schedule | |  |
| 13 | Data extraction Data were extracted using a reporting checklist proposed by the Meta-analysis of Observational Studies in Epidemiology (MOOSE) Group. The searching, data extraction and quality assessment were conducted by two authors independently, and any discrepancies were resolved through iteration and consensus. | |  |
| 14 | Risk of bias (quality) assessment Two review authors will independently assess the risk of bias in included studies. The quality of selected studies for which the full text was available was assessed using the indications developed by the US Department of Health and Human Services specifically for systematic reviews of prognostic tests as follows: 1. Was the study designed to evaluate the new prognostic test, or was it a secondary analysis of data collected for other purposes? 2. Were the subjects somehow referred or selected for testing? What was the testing scenario? 3. Was the clinical population clearly described including the sampling plan, inclusion and exclusion criteria, subject participation, and the spectrum of test results? Did the sample represent patients that would be tested in clinical practice? 4. Did everyone in the samples have a common starting point for follow-up with respect to the outcome of interest including any treatments that could affect the outcome being predicted? 5. Were the prognostic tests clearly described and conducted using a standardized, reliable, and valid method? a. Was the test used and interpreted the same way by all sites/studies including any interdeterminate test results? b. Were the test results ascertained without knowledge of the outcome? c. Were investigators blinded to the test results? d. How were previously established prognostic indicators or other prognostic assessments included in the study and analyses? 6. Was the outcome being predicted clearly defined and ascertained using a standardized, reliable, and valid method? a. How complete was the follow-up of subjects, and were losses to follow-up related to the test results or the outcome being predicted? b. Was the duration of follow-up adequate? 7. Were the data used to develop the prognostic test? a. Were the prognostic groups pre-defined based on clinically meaningful decision thresholds for predicted outcome probabilities? b. Were the results externally validated using an independent sample or internally validated via boot strap or cross-validation methods? c. Were any previously established prognostic indicators or prediction models being used as comparators fit to the sample data in the same manner as the potential new prognostic test? d. Were outcome predictions adjusted for any other factors? Which ones? How? | |  |
| 15 | Strategy for data synthesis We anticipate that there will be limited scope for meta-analysis because of the range of different outcomes measured across the small number of existing trials. However, where studies have used the same type of intervention and comparator, with the same outcome measure, we will pool the results using a random-effects meta-analysis, with standardized mean differences for continuous outcomes and risk ratios for binary outcomes, and calculate 95% confidence intervals and two sided P values for each outcome. In studies where the effects of clustering have not been taken into account, we will adjust the standard deviations for the design effect. Heterogeneity between the studies in effect measures will be assessed using both the? 2 tests and the I2 statistic. We will consider an I2 value greater than 50% indicative of substantial heterogeneity. We will conduct sensitivity analyses based on study quality. We will use stratified meta-analyses to explore heterogeneity in effect estimates according to: study quality; study populations; the logistics of intervention provision; and intervention content. We will also assess evidence of publication bias. | |  |
| 16 | Analysis of subgroups or subsets If the necessary data are available, subgroup analyses will be done for people with different types of urothelial cancer, such as upper urinary tract urothelial cancer, bladder urothelial cancer. | |  |
| 17 | Strategy for data synthesis We anticipate that there will be limited scope for meta-analysis because of the range of different outcomes measured across the small number of existing trials. However, where studies have used the same type of intervention and comparator, with the same outcome measure, we will pool the results using a random-effects meta-analysis, with standardized mean differences for continuous outcomes and risk ratios for binary outcomes, and calculate 95% confidence intervals and two sided P values for each outcome. In studies where the effects of clustering have not been taken into account, we will adjust the standard deviations for the design effect. Heterogeneity between the studies in effect measures will be assessed using both the? 2 tests and the I^2^ statistic. We will consider an I^2^ value greater than 50% indicative of substantial heterogeneity. We will conduct sensitivity analyses based on study quality. We will use stratified meta-analyses to explore heterogeneity in effect estimates according to: study quality; study populations; the logistics of intervention provision; and intervention content. We will also assess evidence of publication bias. | |  |
| 18 | Type and method of review Meta-analysis, Systematic review  Cancer, Neurological, Perioperative care | |  |
| 19 | Language English/Chinese  **Supplemental File 2 PRISMA 2009 checklist**   \| **Section/topic** \| **#** \| **Checklist item** \| **Reported on page #** \| \| --- \| --- \| --- \| --- \| \| **TITLE** \| \| \|  \| \| Title \| 1 \| Identify the report as a systematic review, meta-analysis, or both. \| 1 \| \| **ABSTRACT** \| \| \|  \| \| Structured summary \| 2 \| Provide a structured summary including, as applicable: background; objectives; data sources; study eligibility criteria, participants, and interventions; study appraisal and synthesis methods; results; limitations; conclusions and implications of key findings; systematic review registration number. \| 2 \| \| **INTRODUCTION** \| \| \|  \| \| Rationale \| 3 \| Describe the rationale for the review in the context of what is already known. \| 2-3 \| \| Objectives \| 4 \| Provide an explicit statement of questions being addressed with reference to participants, interventions, comparisons, outcomes, and study design (PICOS). \| 2-3 \| \| **METHODS** \| \| \|  \| \| Protocol and registration \| 5 \| Indicate if a review protocol exists, if and where it can be accessed (e.g., Web address), and, if available, provide registration information including registration number. \| 9/supplemental file 1 \| \| Eligibility criteria \| 6 \| Specify study characteristics (e.g., PICOS, length of follow-up) and report characteristics (e.g., years considered, language, publication status) used as criteria for eligibility, giving rationale. \| 9 \| \| Information sources \| 7 \| Describe all information sources (e.g., databases with dates of coverage, contact with study authors to identify additional studies) in the search and date last searched. \| 9 \| \| Search \| 8 \| Present full electronic search strategy for at least one database, including any limits used, such that it could be repeated. \| 9 \| \| Study selection \| 9 \| State the process for selecting studies (i.e., screening, eligibility, included in systematic review, and, if applicable, included in the meta-analysis). \| 4, 9 \| \| Data collection process \| 10 \| Describe method of data extraction from reports (e.g., piloted forms, independently, in duplicate) and any processes for obtaining and confirming data from investigators. \| 9 \| \| Data items \| 11 \| List and define all variables for which data were sought (e.g., PICOS, funding sources) and any assumptions and simplifications made. \| 9/tables 1-2/ supplemental table 1 \| \| Risk of bias in individual studies \| 12 \| Describe methods used for assessing risk of bias of individual studies (including specification of whether this was done at the study or outcome level), and how this information is to be used in any data synthesis. \| 9-10 \| \| Summary measures \| 13 \| State the principal summary measures (e.g., risk ratio, difference in means). \| Supplemental table 1 \| \| Synthesis of results \| 14 \| Describe the methods of handling data and combining results of studies, if done, including measures of consistency (e.g., I^2^) for each meta-analysis. \| 10 \|  \| **Section/topic** \| **#** \| **Checklist item** \| **Reported on page #** \| \| --- \| --- \| --- \| --- \| \| Risk of bias across studies \| 15 \| Specify any assessment of risk of bias that may affect the cumulative evidence (e.g., publication bias, selective reporting within studies). \| 10 \| \| Additional analyses \| 16 \| Describe methods of additional analyses (e.g., sensitivity or subgroup analyses, meta-regression), if done, indicating which were pre-specified. \| 10 \| \| **RESULTS** \| \| \|  \| \| Study selection \| 17 \| Give numbers of studies screened, assessed for eligibility, and included in the review, with reasons for exclusions at each stage, ideally with a flow diagram. \| 4 \| \| Study characteristics \| 18 \| For each study, present characteristics for which data were extracted (e.g., study size, PICOS, follow-up period) and provide the citations. \| Tables 1-2/Supplemental table 1 \| \| Risk of bias within studies \| 19 \| Present data on risk of bias of each study and, if available, any outcome level assessment (see item 12). \| 4 \| \| Results of individual studies \| 20 \| For all outcomes considered (benefits or harms), present, for each study: (a) simple summary data for each intervention group (b) effect estimates and confidence intervals, ideally with a forest plot. \| 4-5/ Figures 2-3/supplemental figures 1-2 \| \| Synthesis of results \| 21 \| Present results of each meta-analysis done, including confidence intervals and measures of consistency. \| 4-5 \| \| Risk of bias across studies \| 22 \| Present results of any assessment of risk of bias across studies (see Item 15). \| 4/Table 1 \| \| Additional analysis \| 23 \| Give results of additional analyses, if done (e.g., sensitivity or subgroup analyses, meta-regression [see Item 16]). \| 5 \| \| **DISCUSSION** \| \| \|  \| \| Summary of evidence \| 24 \| Summarize the main findings including the strength of evidence for each main outcome; consider their relevance to key groups (e.g., healthcare providers, users, and policy makers). \| 6 \| \| Limitations \| 25 \| Discuss limitations at study and outcome level (e.g., risk of bias), and at review-level (e.g., incomplete retrieval of identified research, reporting bias). \| 7-8 \| \| Conclusions \| 26 \| Provide a general interpretation of the results in the context of other evidence, and implications for future research. \| 8 \| \| **FUNDING** \| \| \|  \| \| Funding \| 27 \| Describe sources of funding for the systematic review and other support (e.g., supply of data); role of funders for the systematic review. \| 14 \|   *From:*  Moher D, Liberati A, Tetzlaff J, Altman DG, The PRISMA Group (2009). Preferred Reporting Items for Systematic Reviews and Meta-Analyses: The PRISMA Statement. PLoS Med 6(7): e1000097. doi:10.1371/journal.pmed1000097  **Supplemental File 3:** **Quality appraisal tool**  **Quality appraisal tool**   \| 1. Was the study designed to evaluate the new prognostic test, or was it a secondary analysis of data collected for other purposes? \| \| --- \| \| 1. Were the subjects somehow referred or selected for testing? What was the testing scenario? \| \| 1. Was the clinical population clearly described including the sampling plan, inclusion and exclusion criteria, subject participation, and the spectrum of test results? Did the sample represent patients that would be tested in clinical practice? \| \| 1. Did everyone in the samples have a common starting point for follow up with respect to the outcome of interest including any treatments that could affect the outcome being predicted? \| \| 1. Were the prognostic tests clearly described and conducted using a standardized, reliable, and valid method?    1. Was the test used and interpreted the same way by all sites/studies including any indeterminate test results?    2. Were the test results ascertained without knowledge of the outcome?    3. Were investigators blinded to the test results?    4. How were previously established prognostic indicators or other prognostic assessments included in the study and analyses? \| \| 1. Were the data used to develop the prognostic test?    1. Were the prognostic groups pre-defined based on clinically meaningful decision thresholds for predicted outcome probabilities?    2. Were the results externally validated using an independent sample or internally validated via boot strap or cross-validation methods?    3. Were any previously established prognostic indicators or prediction models being used as comparators fit to the sample data in the same manner as the potential new prognostic test? \| \| 1. Was the outcome being predicted clearly defined and ascertained using a standardized, reliable, and valid method?    1. How complete was the follow up of subjects, and were losses to follow up related to the test results or the outcome being predicted?    2. Was the duration of follow up adequate? \| \| 1. Were outcome predictions adjusted for any other factors? Which ones? How? \| | |  |
